# Peri-Implant Behavior of Sloped Shoulder Dental Implants Used for All-On-Four Protocols: An Histomorphometric Analysis in Dogs

**DOI:** 10.3390/ma11010119

**Published:** 2018-01-12

**Authors:** Jose Luis Calvo Guirado, Aldo Fabian Lucero-Sánchez, Ana Boquete Castro, Marcus Abboud, Sergio Gehrke, Manuel Fernández Dominguez, Rafael Arcesio Delgado Ruiz

**Affiliations:** 1 Faculty of Health Sciences, Department of Oral and Implant Dentistry, Universidad Católica San Antonio de Murcia (UCAM), 30107 Murcia, Spain; aldo@clinicadea.com (A.F.L.-S.); boket_odo@hotmail.com (A.B.C.); 2 College of Dentistry, Department of Digital Dentistry, University of Kentucky, Lexington, KY 40506-0001, USA; marcus.abboud@gmail.com; 3 Biotecnos Research Center, Rua Dr. Bonazo n 57, 97015-001-Santa Maria (RS), Brazil; sergio.gehrke@hotmail.com; 4 Faculty of Dentistry, Department of Oral and Implant Dentistry, Universidad San Pablo CEU, Grupo HM (Hospital Madrid), 11600 Madrid, Spain clinferfun@yahoo.es; 5 Department of Prosthodontics and Digital Technology, School of Dental Medicine, Stony Brook University, Stony Brook, NY 1103, USA; Rafael.Delgado-Ruiz@stonybrookmedicine.edu

**Keywords:** straight microthread implants, tilted implants, scalloped implants, inmediate loading

## Abstract

The aim of this study was to evaluate the soft tissue thickness and marginal bone loss around dental implants with sloped micro-threaded shoulder (35° angle) in comparing with conventional design, inserted 35° degrees angulated in post extraction sockets and immediate loaded with temporary prosthesis simulating the all-on-four protocol. **Materials and Methods:** Six fox hound dogs received forty-eight post extraction dental implants with the same diameter and length (Medentika, Germany), but with different neck configurations. Two group of implants were inserted 1mm subcrestal. Control group has a micro-threaded neck and the Test group has a sloped microthreaded neck. Immediate loading was applied using a constructed metallic structure. After three months, soft and hard tissue levels were assessed by histomorphometric analysis. **Results:** The mean soft tissue thickness (STT) was 2.5 ± 0.2 mm for the Control group and 3.3 ± 0.3 mm for Test group (*p* = 0.036), meanwhile the mean marginal bone loss (MBL) was 1.53 ± 0.34 mm for Control group and, 1.62 ± 0.22 mm for Test group (*p* > 0.05). **Conclusions:** Within the limitations of this experimental model in dogs, the findings showed that dental implants with microthreaded and microthreaded sloped necks installed in immediate post extraction sites with immediate load, presented a comparable perimplant tissue behavior.

## 1. Introduction

Implant-supported fixed prostheses represent today a common treatment for the rehabilitation of edentulous jaws. The long-established Brånemark protocol recommended the implants to be placed in an upright position, often resulting in a distal cantilever length of even 20 mm [1,2,3] This condition may lead to high bending moments and high stress levels at both the implants and the surrounding bone [4], which in turn, may sustain marginal bone resorption, thus compromising implant survival [5].

The adoption of tilted implants for the rehabilitation of both, edentulous mandibles and maxillae, has been proposed in the recent years. In the mandible, tilting of the distal implants may prevent damage to the mental and mandibular nerve, meanwhile in the edentulous maxilla, the use of implant tilting is an alternative to bone grafting procedures and sinus lift [6]. Implants of conventional length can be placed tilted, allowing for the engagement of as much cortical bone as possible, thus increasing primary stability [7].

Furthermore, increasing the inter-implant distance and reducing cantilever length, a better load distribution may be achieved [8]. Several computational studies suggested the possible biomechanical advantages of implant tilting in full-arch restorations [7,8,9].

Implant tilting associated with immediate loading for the treatment of partial and complete edentulism, especially in the presence of atrophic ridge, is progressively spreading among clinicians. The performance of immediately loaded dental implants supporting partial and full restorations has been evaluated in recent systematic reviews and meta-analyses [10,11,12].

However, immediate implant placement is a challenging surgical procedure and requires proper case selection and surgical technique. Furthermore, there appears to be a lack of clinical guidelines for immediate implant placement case selection.

From a surgical point of view, the insertion of tilted implants implies that enough circumferential bone volume is present to compensate for the periimplant bone resorption after surgery and a prosthetically correct angulation [13,14,15,16]. A perfect prosthetic direction is sometimes impossible because of the morphology of the alveolar crest or the available bone volume. In a recent systematic review, the reduction of the alveolar crest after tooth extraction was calculated as being nearly 4 mm [17].

Novel sloped implant designs were introduced recently in the market to reduce the cantilever extension. The sloped implants might be used for uneven ridges at the anterior and posterior regions. Therefore, a sloped implant configuration could preserve the peri implant soft tissues and marginal bone levels in all of four protocols involving tilted implants. Until the present day, there are not references that explain the biological behavior of sloped micro threaded implants inserted tilted and subject to immediate load animal experiments. Therefore, the aim of this study was to evaluate the soft tissue thickness and marginal bone loss around dental implants with sloped micro-threaded shoulder (35° angle) in comparing with conventional design, inserted 35° degrees angulated in post extraction sockets and immediate loaded with temporary prosthesis simulating the all-on-four protocol. 

## 2. Results

The mean and SD of soft tissue thickness (STT) and the mean and SD of marginal bone loss (MBL) were expressed in Table 1 and Table 2.

In relation to marginal bone loss, similar results were obtained for the group of implants placed in an axial position (Control) when compared to tilted implants (Test). Marginal bone loss values were low in all of the cases, and there were no significant differences between Controls and Test (Table 2).

## 3. Discussion

Is frequent to find atrophic maxillae were the insertion of implants is compromised. In order to avoid complex augmentation procedures, different authors have preconized the insertion of tilted implants [6,7,8,9]. Bellini et al. concluded that the insertion of 30° tilted implants may lead to a higher reduction of stress values at the bone-to-implant interface when compared with non-tilted implants [8].

Moreover, current literature shows that immediate loading of dental implants presents high implant survival and success rates, so it is gaining recognition as suitable for most patients in need of full jaw rehabilitation [18]. Testori et al. concluded that immediate loading associated with tilted implants is a valid treatment modality for atrophic maxilla [19]. Malò et al. in a retrospective study including 176 immediately loaded implants with fixed prostheses in edentulous mandibles via an “All-on-four” concept, showed minimal marginal bone resorption in all of the implants [20].

In this sense, the meta-analysis carried out by Monje et al. assessed that marginal bone loss around tilted implants splinted to support a fixed prothesis was not significantly different in comparison to straight implants [21]. In the same way, Koutouzis & Wennström carried out a longitudinal retrospective study and concluded that implant inclination does not significantly influence periimplant bone resorption after five years of loading [22]. Menini et al. carried out a systematic review and concluded that the immediate loading of tilted implants is a predictable treatment option, however, authors remarked the necessity to establish what is the minimum angulation that is required to define an implant as tilted [23].

Barnea et al. carried out a retrospective study to analyze the influence of implant angulation on marginal bone loss. Authors analyzed twenty-nine patients with freestanding FPDs supported by two implants. The anterior implant was placed axially, and the posterior was tilted distally. The authors compared the average bone loss between straight and tilted implants. Average bone loss after one year was 0.89 for axial implants, and 0.98 for tilted implants. They found there was no significant correlation between implant angulation and bone loss [24].

Related to marginal bone loss, similar results were obtained for the group of implants placed in an axial position (Control) when compared to tilted implants (Test) with no significant differences between Controls and Test.

In another recent publication, Chercanovic et al. carried out a meta-analysis and did not find significant differences between tilted dental implants on the occurrence of greater marginal bone loss in comparison with axially placed implants, which is in accordance with our results [25].

In this sense, a recent systematic review carried out by Ata-Ali et al. concluded that there are no differences in success rates between axial and tilted implants. Moreover, the marginal bone loss was similar for tilted and axial implants in the prospective and retrospective studies subjected to review. The authors assessed that tilted implants exhibit the same behavior as implants that were placed in an axial position, which is in accordance with the results obtained in our study [26].

In relation to soft tissues, there are not any published studies regarding this aspect in relation to insertion of tilted implants. In the literature, we only find the study of Krennmair et al. in which the authors analyzed pocket depth around the implants; they did not find significant differences in peri-implant probing depth between axial or tilted implants [27].

In our study, values recorded for soft tissue thickness were higher for tilted implants and differences were statistically significant in comparison with implants placed axially (*p* < 0.05*) (Table 2), thus favoring the hygiene of the implants and contributing to the long-term success of implants and prostheses.

## 4. Materials and Methods

Six fox hound dogs of approximately one year of age, each weighing approximately 14–15 kg were used in the experiment. The animals were fed with a daily pellet diet. Veterinarian clinical examination determined that the dogs were in good general health. The protocol of this animal study was approved by the Ethics Committee for Animal Research at the University of Murcia, Spain, following the guidelines established by the European Union Council Directive Royal Decree 53/2013, of February 1 (2010/63/UE), by laying down the basic rules for the protection of animals used for experimental and other scientific purposes, including teaching.

**Figure 1:**
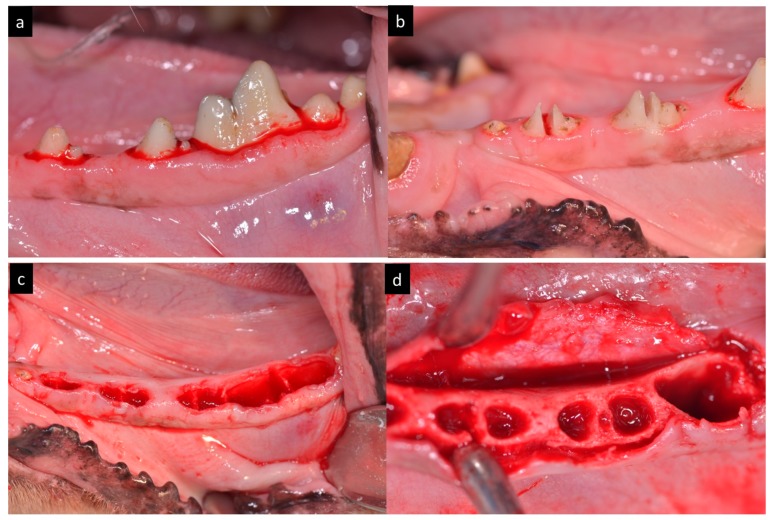
(**a**) Preoperative aspect of the teeth. (**b**) Teeth sectioned in bucco-lingual direction. (**c**) Post extraction sockets. (**d**) Full-thickness flap.

The animals were pre-anaesthetized administering a premedication combination of 0.1 mg/kg of intramuscular acepromazine and 0.2 mg/kg of intramuscular butorphanol, followed by induction with 3.3 mg/kg of intravenous propofol. The mixture was injected intramuscularly in the femoral quadriceps, an intravenous catheter was inserted into the cephalic vein. Additionally, a conventional dental infiltration anaesthesia was administered from mesial of the second lower premolar to distal of the first lower molar bilaterally. The teeth were sectioned in a buccolingual direction at the bifurcation level using a carbide bur. Afterwards, the roots were extracted using periotome and forceps without damaging the bony walls. Mandibular premolars and first molar (P_2_, P_3_, P_4_, M_1_) were extracted in both hemi-arches of each dog (Figure 1).

**Figure 2:**
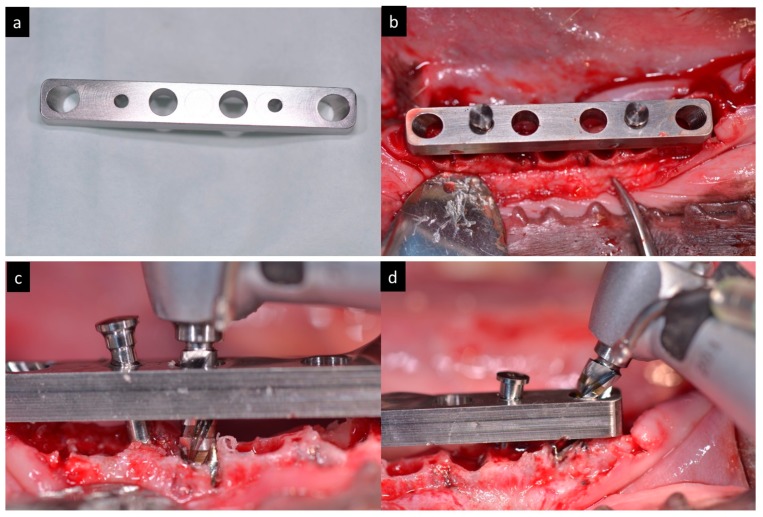
(**a**) Aspect of the surgical guide. (**b**) Surgical guide fixed in the mandible. (**c**) Drilling trough the surgical guide in axial direction. (**d**) Drilling trough the surgical guide in tilted direction.

Surgical guide with four orifices (two axial 0° degrees and two inclined in 35° degrees were anchored with intraosseous pins to the cortical bone and a guided drilling protocol was used for the osteotomies (Figure 2).

**Figure 3:**
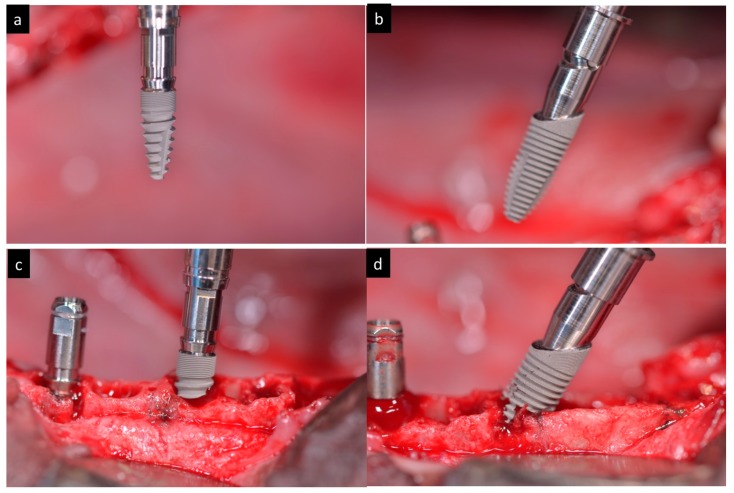
(**a**) Macroscopically aspect of the axial implant. (**b**) Macroscopically aspect of the sloped implant. (**c**) Insertion of an axial implant. (**d**) Insertion of a sloped implant.

Forty-eight Medentika™ dental implants (Medentika GmbH, Hügelsheim, Germany) with 3.5 mm diameter and 9 mm length with two different designs were inserted inside extracted distal roots (both straight and sloped implants), divided in two groups (*n* = 24 per group): the Control group used a conventional implant design with axial micro threads; the Test group used sloped implants with 35° degrees angle and micro threads (Figure 3).

**Figure 4:**
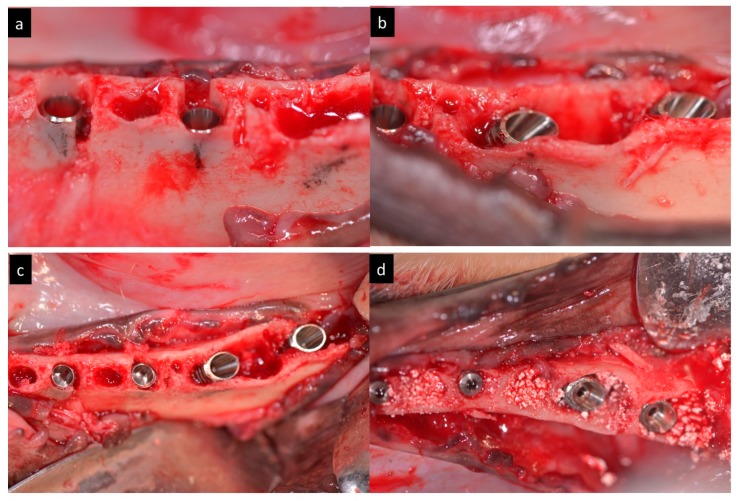
(**a**–**d**) Clinical view of the immediate implants inserted 1 mm subcrestally in distal roots.

The implants were submerged with their platforms located 1 mm sub crestal (Figure 4).

**Figure 5:**
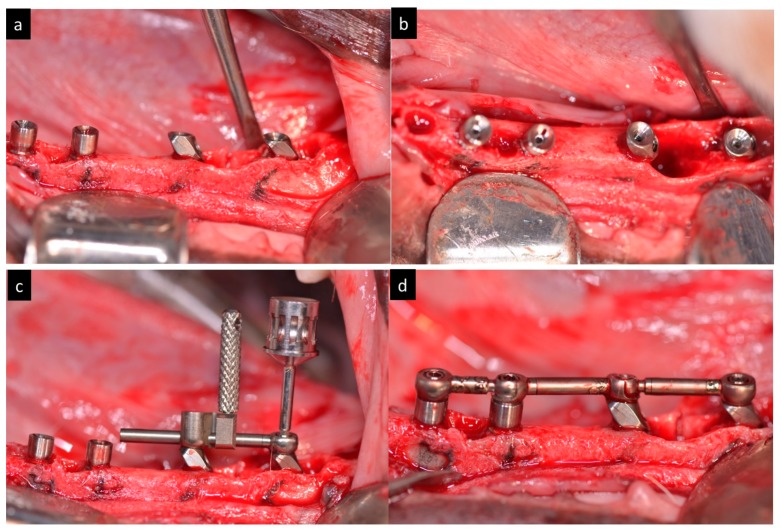
(**a**) Lateral view of abutments connected to the implants. (**b**) Oclusal view of abutments connected to the implants. (**c**) Screwing of the bar. (**d**) Clinical aspect of the bar framework screwed in the mandibles.

Afterwards, the implants were splinted with a bar framework system and a temporary acrylic restoration was fabricated to perform immediate load (Figure 5). The occlusion was adjusted to have at least three contacts per side. 

During the first week, the animals received antibiotics (Amoxicillin 500 mg) and analgesics (Ibuprofen 600 mg) (three times a day) via the systemic route. Resorbable sutures were removed after two weeks. The dogs were fed with a soft diet for 14 days.

Healing was evaluated weekly and plaque control was maintained by flushing the oral cavity with SEA4 Total care (Blue Sea Laboratories, Alicante, Spain) weekly during the whole experimental period.

After three months, the animals were sedated and anesthetized following the previous procedure and euthanized with an overdose of Pentothal Natrium (Abbott Laboratories, North Chicago, IL, USA).

### 4.1. Histological Processing

Biopsies were processed according to the methods described by Donath and Breuner (1982). In brief, the samples were dehydrated in increasing grades of ethanol up to 100%, infiltrated with methacrylate, polymerized, and sectioned at the medial-distal plane using a diamond saw (Exakt, Apparatebau, Norderstedt, Germany). Two sections of 100 µm thickness were obtained containing the implant and surrounding tissues. Each section was ground up to a final thickness of 40 µm and stained using toluidine blue stain.

### 4.2. Histomorphometric Analysis

Histomorphometric analysis was performed using calibrated digital images at ×10 magnification (Leica microscope Q500Mc, Leica DFC320s, 3088 × 2550 pixels, Leica Microsystems, Barcelona, Germany). The most central sagittal section of each implant was taken for the histomorphometric analysis using MIP 4.5 software (Microns Image Processing Software, CID, Consulting Image Digital, Barcelona, Spain) connected to a Sony DXC-151s 2/3-CCD RGB Color Video Camera.

### 4.3. Soft Tissue Thickness

**Figure 6:**
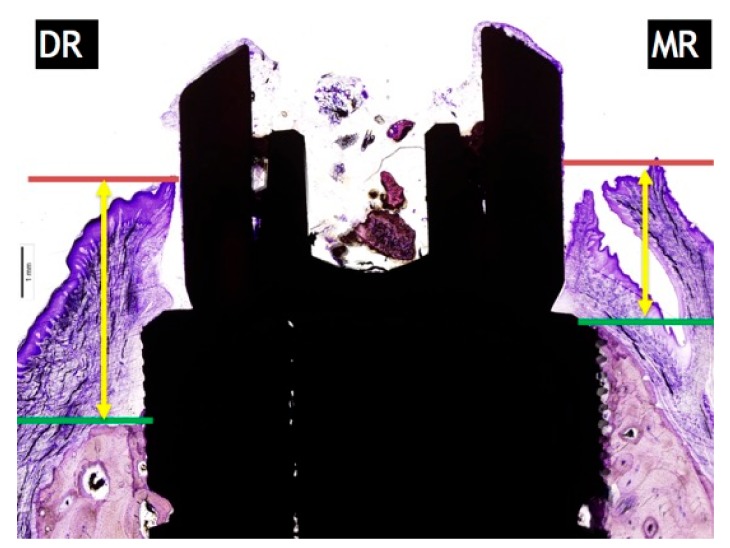
Measurements of soft tissue thickness: DR: distal resorption (distance from the top of the implant shoulder to the first BIC in the distal side. MR: mesial resorption (distance from the top of the implant shoulder to the first BIC in the mesial side).

The soft tissue thickness was measured from the most higher point of the gingival mucosa to the most coronal point of the crestal bone. The measurements of buccal and lingual mean and SD were expressed in millimetres (Figure 6).

### 4.4. Marginal Bone Loss

**Figure 7:**
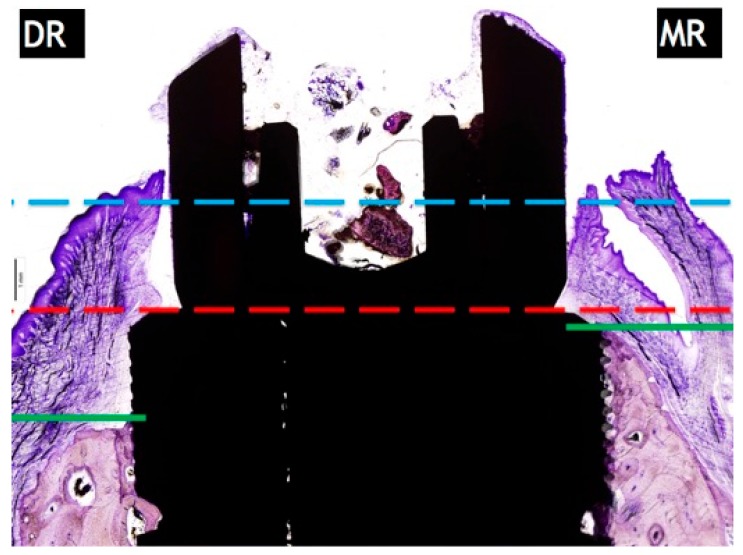
Measurement of marginal bone loss: DR: distal resorption (distance from the top of the implant shoulder to the first BIC in the distal side. MR: mesial resorption (distance from the top of the implant shoulder to the first BIC in the mesial side).

The distance from the implant platform to the highest portion of the mesial and distal bone. The measurements of buccal and lingual crests bone loss were expressed in millimetres (Figure 7). To facilitate differentiation between native and newly formed bone, blue and light blue chromaticity were enhanced by digital processing. Mesial bone wall resorption in relation to the distal bone wall was expressed as a linear measurement in millimeters (relative measurement).

### 4.5. Statistical Analysis

Mean values and standard deviations were calculated. The mean differences between the groups for each histomorphometric parameter were analysed using the Student’s *t*-test. The dog was the unit of measure. The level of significance was set as *p* < 0.05.

## 5. Conclusions

Within the limitations of this experimental study in dogs can be concluded that:

The soft tissue thickness of micro threaded and sloped micro threaded implants is significantly higher when the implants are tilted. Differences in the axial (0° degrees) and tilted angulation (35° degrees) of micro threaded and sloped micro threaded dental implants produces similar marginal bone loss. The insertion of the new sloped implants is a valid treatment option to combine with immediate loading protocols.

## Figures and Tables

**Table 1:** Soft tissue thickness (STT) comparison between axial and tilted implants. The group B implants showed more STT when compared to group A implants. Significant difference was *p* < 0.05.

Soft Tissue Thickness (STT) mm	Control Axial Implants	Test Tilted Implants
Mean ± SD	2.5 ± 0.2 mm	3.6 ± 0.3 mm
*p* value		*p* = 0.036

**Table 2:** Marginal bone loss (MBL) comparison between axial and tilted implants. The groups A and B implants showed similar MBL without significant differences *p* > 0.05.

Marginal Bone Loss (MBL) mm	Group A Axial Implants	Group B Tilted Implants
Mean ± SD	1.53 ± 0.34 mm	1.62 ± 0.22 mm
*p* value		*p* = 0.872
