# Correction: Calvo Guirado, J.L., et al. Peri-Implant Behavior of Sloped Shoulder Dental Implants Used for All-On-Four Protocols: An Histomorphometric Analysis in Dogs. *Materials* 2018, *11*, 119

**DOI:** 10.3390/ma11091614

**Published:** 2018-09-04

**Authors:** José Luis Calvo Guirado, Aldo Fabian Lucero-Sánchez, Ana Boquete Castro, Marcus Abboud, Sergio Gehrke, Manuel Fernández Dominguez, Rafael Arcesio Delgado Ruiz

**Affiliations:** 1 Faculty of Health Sciences, Department of Oral and Implant Dentistry, Universidad Católica San Antonio de Murcia (UCAM), 30107 Murcia, Spain; aldo@clinicadea.com (A.F.L.-S.); boket_odo@hotmail.com (A.B.C.); 2 College of Dentistry, Department of Digital Dentistry, University of Kentucky, Lexington, KY 40506-0001, USA; marcus.abboud@gmail.com; 3 Biotecnos Research Center, Rua Dr. Bonazo n 57, 97015-001-Santa Maria (RS), Brazil; sergio.gehrke@hotmail.com; 4 Faculty of Dentistry, Department of Oral and Implant Dentistry, Universidad San Pablo CEU, Grupo HM (Hospital Madrid), 11600 Madrid, Spain; clinferfun@yahoo.es; 5 Department of Prosthodontics and Digital Technology, School of Dental Medicine, Stony Brook University, Stony Brook, NY 1103, USA; Rafael.Delgado-Ruiz@stonybrookmedicine.edu

The authors would like to correct following typing and two images errors:

In “Abstract”, authors change 35° to 30°, and change the word “same” to “different”. The correct expressions are given as:

**Abstract:** The aim of this study was to evaluate the soft tissue thickness and marginal bone loss around dental implants with sloped micro-threaded shoulder (30° angle) in comparison to conventional design, inserted 30° angulated in post extraction sockets and immediately loaded with temporary prosthesis simulating the all-on-four protocol. **Materials and Methods:** Six fox hound dogs received forty-eight post extraction dental implants with the different diameter…

In “4. Materials and Methods”, authors change 35° to 30°, “Medentika™” to “Quattrocone Medentika”, “with 3.5 mm diameter and 9 mm length” to “with 3.5 mm diameter by 9 mm length for Quattrocone straight implants and 4.3 mm diameter by 9 mm length for Quattocone30 dental implants”.

The correct expressions are given as:

Surgical guide with four orifices (two axial (0°) and two inclined at 30°) were anchored with intraosseous pins to the cortical bone and a guided drilling protocol was used for the osteotomies (Figure 2).

Forty-eight Medentika™ dental implants (Medentika GmbH, Hügelsheim, Germany) with 3.5 mm diameter by 9 mm length for Quattrocone straight implants and 4.3 mm diameter by 9 mm length for Quattrocone30 dental implants with two different designs were inserted inside extracted distal roots (both straight and sloped implants), divided into two groups (n = 24 per group): the Control group used a conventional implant design with axial micro threads; the Test group used sloped implants with 30° angle and micro threads (Figure 3).

In “4.1. Histological Processing”, authors change “medial-distal plane” to “buccal-lingual plate”

The correct expressions are given as:

Biopsies were processed according to the methods described by Donath and Breuner (1982). In brief, the samples were dehydrated in increasing grades of ethanol up to 100%, infiltrated with methacrylate, polymerized, and sectioned at the buccal-lingual plane using a diamond saw (Exakt, Apparatebau, Norderstedt, Germany)…

The new image of Figure 6 is as follows:

In “4.4. Marginal Bone Loss”, authors change “mesial and distal bone” to “buccal and lingual bone”, “Mesial bone Wall” to “Buccal bone Wall”, “distal bone Wall” to “lingual bone Wall”

The correct expressions are given as:

The distance from the implant platform to the highest portion of the buccal and lingual bone. The measurements of buccal and lingual crests bone loss were expressed in millimeters (Figure 7). To facilitate differentiation between native and newly formed bone, blue and light blue chromaticity were enhanced by digital processing. Buccal bone wall resorption in relation to the lingual bone wall was expressed as a linear measurement in millimeters (relative measurement).

The new image of Figure 7 is as follows:

In “5. Conclusions”, authors change 35° to 30°. The correct expressions are given as:

Within the limitations of this experimental study in dogs can be concluded that:

The soft tissue thickness of micro threaded and sloped micro threaded implants is significantly higher when the implants are tilted. Differences in the axial (0°) and tilted angulation (30°) of micro threaded and sloped micro threaded dental implants produces similar marginal bone loss. The insertion of the new sloped implants is a valid treatment option to combine with immediate loading protocols.

The authors would like to express their gratitude to Ana Boquete-Castro and Aldo Lucero for finding these errors.

The changes do not affect the results. The manuscript will be updated, and the original one will remain available on the article webpage.

## Figures and Tables

**Figure 6:**
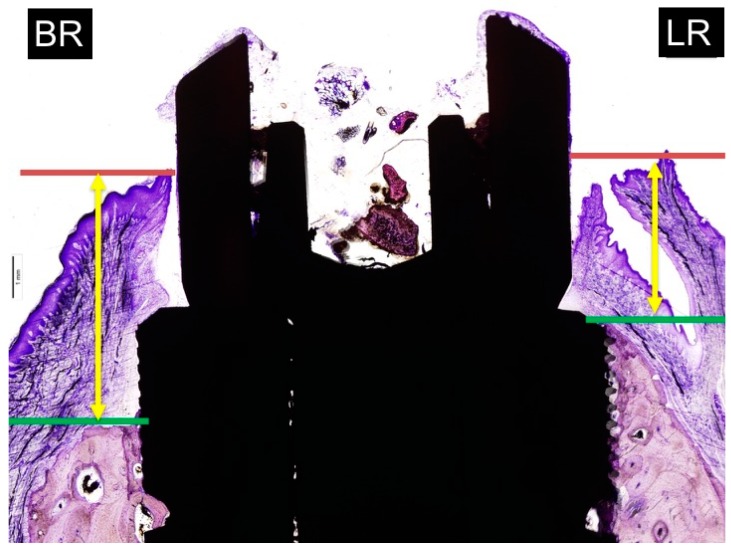
Measurements of soft tissue thickness: BR: buccal crest resorption (distance from the top of the implant shoulder to the first BIC in the buccal crestal side. LR: lingual crest resorption (distance from the top of the implant shoulder to the first BIC in the lingual crestal side).

**Figure 7:**
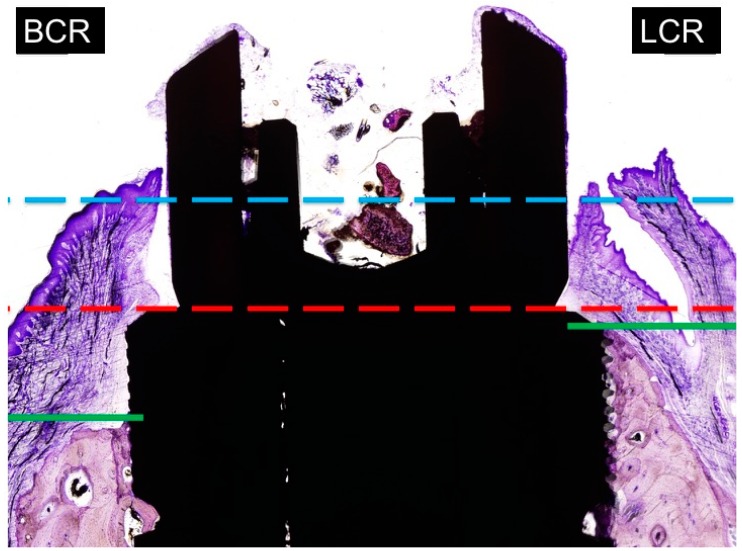
Measurements of marginal bone loss: BCR: buccal crest resorption (distance from the top of the implant shoulder to the first BIC in the buccal crest side. LCR: lingual crest resorption (distance from the top of the implant shoulder to the first BIC in the lingual crest side).

